# Successful Pregnancy of a 39-Year-Old Woman After Endometrial Infection During In Vitro Fertilization Treatment: A Case Report

**DOI:** 10.7759/cureus.49476

**Published:** 2023-11-27

**Authors:** Priti Karadbhajne, Akash More

**Affiliations:** 1 Clinical Embryology, Jawaharlal Nehru Medical College, Datta Meghe Institute of Higher Education and Research, Wardha, IND

**Keywords:** implantation, lactobacillus, genital tract, microbes, endometrium

## Abstract

The female reproductive system shows the presence of some microbial species. In in vitro fertilization (IVF) treatment, a healthy endometrium plays a key role in embryo implantation, but in case of endometrial infection, it is proven as an obstacle for IVF treatment if not identified. Microorganisms from the genital tract have significant consequences for the reproductive tract health and infection susceptibility.

This case study assesses the intricate connection between endometrial microbiota and successful embryo implantation with IVF after endometrial infection. The report describes the case of a 39-year-old female with secondary infertility who was struggling to conceive for the last 18 years. The patient was suffering from hypothyroidism. Her thyroid was controlled using tablets up to a normal value 2.97 of µIU/ml. Hormonal profile of the patient was normal according to her age for IVF. After intra-cytoplasmic sperm injection, three embryos were formed. However, due to endometrial infection, embryo transfer was delayed, and infection was treated with medications. After complete removal of infection, embryo transfer was successful, achieving a positive result in pregnancy.

This study underlines the need for intended infertility treatments and highlights the potential role of endometrial bacteria in reproductive success.

## Introduction

Microbial entities originating from the genital system exhibit a robust correlation with the overall health status and susceptibility to infections within the reproductive tract. Notably, *Lactobacillus* species have been substantiated as effective barriers against infections and wield significant influence in molding the composition of microorganisms within the vaginal microbiome [1]. Controversy surrounds the existence of a diverse array of microorganisms within the female reproductive system beyond the confines of the vaginal environment. The vaginal microflora actively modulates the pH levels, maintaining them within the range of 3.5 to 4.5 through the production of lactic acid, which is imperative in thwarting the proliferation of potentially pathogenic bacteria [2]. This intricate ecosystem hosts a multitude of bacteria, accounting for approximately 9% of the microbial presence within the reproductive tract, although not all can be readily cultured under laboratory conditions. In 2002, the seminal discovery of nonculturable bacteria was marked using molecular techniques [3].

In some articles, researchers mentioned the evidence that points out the detrimental impact of bacterial presence during embryo transfer on clinical pregnancy. The presence of bacteria was confirmed through microbiological analysis of patient's transfer catheter tip used in in vitro fertilization (IVF) procedure. This presence of microbes has been directly related to the failure or success of embryo implantation [3,4]. Researchers delineate a classification of microorganisms into two distinct categories: *Lactobacillus*-dominant (LD) and non-*Lactobacillus*-dominant (NLD) bacteria [5]. A preponderance of these bacterial entities is ubiquitously distributed both within the vaginal milieu and the endometrial environment. Investigations into the stratified microbial compositions of biofilms along the reproductive tract have primarily focused on the microbial communities within the upper genital tract. Remarkably, these biofilms, frequently encountered within the vaginal habitat, have also been ascertained within the endometrial cavity and extending into the fallopian tubes [6].

The focal point of this case report resides in elucidating the intricate correlation between the microbial community inhabiting the endometrial environment and the consequential triumph of embryo implantation during IVF procedures after instances of endometrial infection.

## Case presentation

A 39-year-old patient was enrolled in 2021 at Wardha Test Tube Baby Centre (WTTBC), Sawangi (Medhe), Wardha, Maharashtra, India, with a complaint of infertility. The couple had been facing infertility for 18 years. They were registered for three IVF cycles, including three ovum pickup (OPU) and three embryo transfers at WTTBC. A 39-year-old lady was married 20 years ago. The patient was an engineer by profession and working in a software company. Written consent was taken from them. No addiction was found in the couple, such as tobacco or liquor consumption on a daily basis.

Family history related to genetic disorder

The patient had no family history of diabetes, blood pressure, tuberculosis, bronchitis, or surgeries. There were no findings of psychological problems in both families.

Medical history of the couple

This case report describes a female patient who was affected by secondary infertility. Ultrasound findings revealed the presence of a healthy uterus. Her menarche started at 15 years of age. The menstrual cycle was irregular, with a four to five months’ gap. Blood loss was for four days on average. The patient was suffering from hypothyroidism, which was controlled by taking medication. Laparoscopic ovarian cystectomy was performed in 2015. Her blood group was B +ve.

According to the semen analysis report of the husband, sperm count was 60 mil/mL and motility was 50%. Morphological defects of sperm were analyzed, and it was 97%. Only 3% of normal morphology having sperms were available for ICSI. The interpretation of the analysis report was teratozoospermia. His blood group was also B +ve. The patient had a history of intrauterine insemination (IUI) failure six times with the husband's semen.

Clinical findings

Hormone investigation, such as thyroid-stimulating hormone (TSH), anti-mullerian hormone (AMH), prolactin, luteinizing hormone (LH), and follicle-stimulating hormone (FSH), plays a key role in IVF treatment. Ovarian reserve can be estimated from the value of AMH. AMH should be between 2 and 6.8 ng/mL to project a healthy ovarian reserve. It helps the clinician to initiate the IVF treatment. FSH and LH ratio is useful in predicting IVF cycle results before the initiation of stimulation. According to some researchers, an increased level of prolactin in follicular fluid is positively related to the receptivity ability of endometrium, the existing ability of sperm with slow motility, and a rise in oocyte efficacy.

In our case study, the hormonal profile of a female patient was observed as follows: AMH value was 2.08 ng/mL. Serum LH and FSH values were 15.07 mIU/mL and 5.92 mIU/mL, respectively. The value of estradiol (E2) was 1873 pg/mL before OPU. Prolactin hormone was measured at 8.35 ng/mL. Ultra TSH value was 2.97 µIU/ml, indicating a normal level.

**Figure 1:**
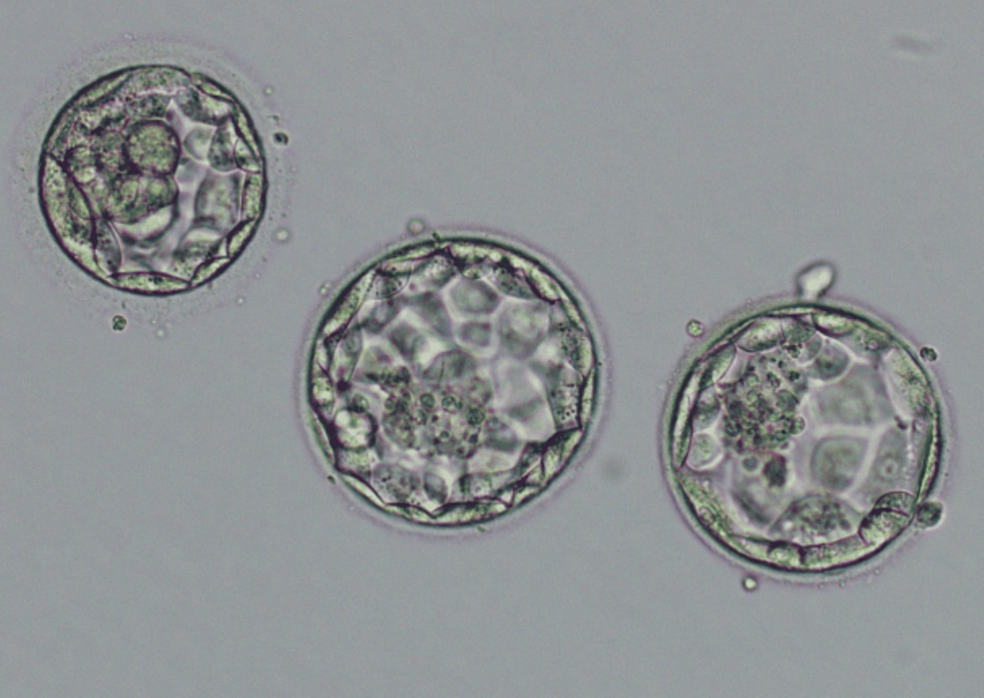
Embryo of grades 4AA, 3AA, and 3AB transferred to the patient

Antagonist protocol was used by the clinician for IVF stimulation procedure of the patient. A patient was stimulated with recombinant follicle-stimulating hormone (rFSH) 150 IU/day from day 2 of the cycle. A dose of rFSH will fluctuate up to 300 IU/day and continue with follicle size and number observed in an ultrasound scan. Follicle size will be 18 mm up to ovulation day with a fluctuating dose of rFSH. On day 10, human chorionic gonadotropin (HCG) 10,000 IU was injected as a trigger for final oocyte maturation. Oocyte retrieval was performed on day 12 of stimulation. On ovulation day, 10 follicles were retrieved in follicular fluid. Among them, seven matured oocyte (MII) was used for ICSI. After five days, three blastocysts of 4AA, 3AA, and 3AB grades were formed (Figure 1). (Embryo grades are given according to the embryo grading system.) These embryos were cryopreserved in a liquid nitrogen storage can. The clinician planned for frozen embryo transfer after two months.

The patient came to the clinic after two months for embryo transfer preparation on day 2 of menses. The patient complained of fluid discharge and itching. The clinician noted endometrial scratching after screening of the vagina and sent an endometrial tissue sample to the laboratory for a culture sensitivity test. A culture sensitivity test was found positive for *Pseudomonas*. The clinician prescribed a combination therapy of gentamycin and levofloxacin content tablets. Levofloxacin acts as an inhibitor for bacterial deoxyribonucleic acid (DNA) synthesis, and it is proven effective against *Pseudomonas* species bacteria. The drug content is fluoroquinolone.

After one month, the clinician again sent a sample for a culture sensitivity test, and it was negative. The clinician started embryo transfer preparation for the patient after complete recovery. Embryo transfer preparation includes tablet estrogen 2 mg daily and multivitamin tablet consumption. Embryo thawing was done early in the morning using a thawing kit, and embryos were kept in a planner incubator for 2 hours. Embryo transfer was done after 2 hours, and three embryos were transferred. Blood sample was sent to the laboratory after 14 days for beta-hCG value. The clinical pregnancy test was positive after 14 days. The clinician prescribed some medications after a positive clinical pregnancy test and suggested that the patient visit the clinic for regular follow-up to delivery.

## Discussion

Moreno et al. supported the theory of presence of microorganism in the endometrium and dissociated it from hormone modulation. The presence of non-lactobacillus microbes in the endometrium is directly related to the negative outcome of IVF. The findings reported to broaden the assessment of endometrial receptivity from a microbiological perspective in addition to the morphological and molecular levels. In reproductive medicine, it is time to view microbes as friends as well as threats. Due to this classification, recognition of endometrial health became possible, and they correlated it with the clinical outcome [3].

Giudice reported that implantation depends on various factors for a successful result and is a complex process, and supports the theory suggested by Moreno et al. [4].

Franasiak et al.’s study used technology-related sequencing. They used the tissue material collected from the embryo transfer catheter tip for amplification and avoided to extract fluid as sample. They described how to collect the sample and examine it and how to differentiate the microbes [5].

Hashimoto and Kyono analyzed 99 female patients for this study. Patient history, microbial infection, clinical pregnancy, and lactobacilli percentage in the endometrium were examined. They found 68 cases with healthy endometrium and 31 cases with endometrium with microbial infection. This categorization made it possible to diagnose IVF patients' endometrial microbiological health and determine how it related to their reproductive success [6].

Kitaya et al. analyzed patients with three different types of cycle including natural, hCG-triggered, and hormone replacement cycles. They included patients with recurrent implantation failure and investigated two kinds of samples, vaginal secretion (VS) and endometrial fluid (EF). Some differences between VS and EF were observed for microbes in the same patient [7].

Some studies reported that the stability of endometrial microbes was achieved in the luteal phase during procurement of the receptive state of the endometrium. Kyono et al. stated that their study differed from other studies in terms of origin of patients and time of sample collection. According to them, this study is based on microbes of the endometrium and vagina in infertile patients and healthy patients. There was difference in the percentages of Lactobacilli in the endometrial microbiome of healthy and infertile patients. They concluded that rise in the lactobacilli percentage might be successful for implantation in infertile patients [8].

In a study by Liu et al., the microorganisms of fluid are not equivalent to endometrial tissue. A complete view of microbial colonization is possible with sampling from both EF and biopsies [9]. Most of the studies have limited the sampling numbers of patients and different sites of swab and tissue collection. Some studies described the molecular biology method for detecting pathogenic and non-pathogenic microorganisms. Still, the molecular method will be expensive, and it is not affordable for rural residential IVF patients [10]. Wee et al. described in their study the comparison between infertile patients and healthy females to investigate the microorganism of the vagina, cervix, and endometrium. Endometrial microbiota will likely differ from the lower reproductive tract in small but significant ways. The study suggests the future proposition of endometrial microorganisms [11].

Therapeutic therapies that address endometrial dysbiosis or augment endometrial receptivity might evolve, improving IVF outcomes [12]. Our case report provides conclusive proof that the endometrial microbiota exhibits discernible, albeit nuanced, variations from the microbial composition of the lower reproductive tract. Additionally, it highlights the prospective significance of the microbiota in the lower reproductive tract, positing its influence on the etiology of infertility. In addition, this work emphasizes the need for large-scale studies to investigate the complex interactions between microbial communities, reproductive health, and assisted reproductive technology.

This study includes the endometrial infection, treatment during IVF, and successful clinical pregnancy of the infertile patient. Further research is needed for the investigation of the mechanism of pathogenicity of microbes and how it will affect clinical outcomes.

## Conclusions

This study described the journey of a 39-year-old woman with endometrial infection during IVF treatment and successful clinical outcome. This case underscores the importance of customized techniques in addressing infertility issues and the potential impact of the endometrial microbiome on implantation success. This accomplishment highlights patients' tenacity and the growing environment of assisted reproductive procedures, providing hope for individuals navigating the challenges of fertility treatments.
